# Effects of Integrated Neuromuscular Training on Physical Fitness in Badminton Athletes of Different Maturity Statuses

**DOI:** 10.3390/children12070830

**Published:** 2025-06-23

**Authors:** Ming-Chia Weng, Xiang Dai, Chih-Hui Chiu, Chien-Chang Ho, Chia-Cheng Liu, Shuo-Min Hsu, Che-Hsiu Chen

**Affiliations:** 1 Department of Physical Education, Chinese Culture University, Taipei 11114, Taiwan; omegaweng1108@gmail.com; 2 Department of Sport Performance, National Taiwan University of Sport, Taichung 404401, Taiwan; fg00967@yahoo.com.tw; 3 Graduate Program in Department of Exercise Health Science, National Taiwan University of Sport, Taichung 404401, Taiwan; chiuch@gm.ntus.edu.tw; 4 Department of Physical Education, Fu Jen Catholic University, New Taipei City 242062, Taiwan; 093703@mail.fju.edu.tw; 5 Sports Medicine Center, Fu Jen Catholic Hospital, New Taipei City 242062, Taiwan; 6 Department of Ball Sports, National Taiwan University of Sport, Taichung 404401, Taiwan; yonex624@gm.ntus.edu.tw; 7 Department of Family medicine, Show Chwan Memorial Hospital, Changhua 50544, Taiwan; u103022415@cmu.edu.tw

**Keywords:** strength, knee, overhead athlete, core stabilization

## Abstract

**Background/Objectives:** Dominant leg use in badminton may contribute to lower limb asymmetry, potentially affecting performance and injury risk. This study investigated the effects of a 12-week integrated neuromuscular training (NMT) program on sports performance. **Methods:** Twenty-four well-trained male badminton players (age: 13.5 ± 1.15 years) were randomly assigned to groups based on maturation status (pre-peak height velocity [pre-PHV] and post-peak height velocity [post-PHV]; *n* = 12 each). All participants completed two NMT sessions weekly. Pre- and post-training assessments included a 20 m sprint, countermovement jump (CMJ), agility *t*-text, hexagon test, and Y-balance test. **Results:** Both groups improved significantly across most tests. The post-PHV group (ES: 0.70–1.35) showed greater improvements in sprinting, CMJ, and agility, while the pre-PHV group (ES: 0.39–1.23) improved more in balance and asymmetry. **Conclusions:** These results underscore the need for age- and maturity-specific training strategies to optimize performance and address asymmetries in youth athletes.

## 1. Introduction

Badminton is one of the most widely enjoyed sports worldwide, with approximately 200 million individuals engaging in the activity [1]. The sport involves rapid, multidirectional footwork, as well as jumping and lunging movements [2]. Research on adolescent badminton players indicates a notably high incidence of stress fractures in the lower leg following long-term training. The most frequently injured areas include the lower back, knees, hips, and ankles [3]. Over time, badminton players often develop weaker knee flexors, imbalanced muscle strength between flexors and extensors, decreased lower limb stability, and a reduced symmetry index between the dominant and non-dominant sides [4].

Adolescence is categorized into early adolescence (aged 11–14 years) and late adolescence (aged 15–19 years), with the distinction based on chronological age. The peak growth velocity period, also known as the peak height velocity (PHV) or “growth spurt,” typically occurs around ages 10–12 for females and 12–14 for males. For adolescents, the primary goal of neuromuscular training (NMT) is to enhance athletic performance [5]. Integrated NMT exercises usually include strength training, coordination exercises, balance control training, plyometrics, agility, and flexibility [6,7,8,9,10]. These modalities have been empirically shown to improve biomechanical movement patterns and sports performances and reduce injury risk, provided that they are implemented under the supervision of qualified coaches who design and oversee the training programs [11,12].

Recent studies suggest that categorizing athletes by their peak height velocity (PHV)—into pre-PHV and post-PHV groups—may offer a more individualized and effective training approach. For example, a four-week (two sessions per week) multicomponent NMT program significantly improved squat stability and enhanced trunk and hip joint alignment in elite adolescent cricket players categorized by PHV stage [13]. Similarly, a six-week change-of-direction training program, performed twice weekly for 20–25 min per session, led to improvements in jump performance, balance, and sprint ability in both pre- and post-PHV elite adolescent soccer players [14]. Furthermore, an eight-week resistance training intervention in adolescent swimmers showed positive trends in maximal isometric pull-up strength for both pre- and post-PHV groups, with greater improvements observed in post-PHV athletes [15]. These findings suggest that strength and conditioning coaches, as well as sports trainers, should recognize the importance of tailoring training programs according to an athlete’s maturation stage—specifically, the pre-PHV and post-PHV phases [16].

Notably, there is a lack of research on the long-term effects of integrated NMT on youth badminton players across different developmental stages. Only one study focusing on female badminton players (aged 16–19 years) demonstrated that 8 weeks of integrated NMT effectively improved functional movement screening scores, vertical jump, balance ability, strength, and overall athletic performance [17]. Therefore, the aim of this study is to investigate the effects of integrated neuromuscular training on lower body speed, jump, agility, and balance performance in male badminton players with different maturation statuses.

## 2. Methods

### 2.1. Experimental Approach to the Problem

This study aimed to evaluate the effects of integrated NMT on lower extremity sports performance in pre-PHV and post-PHV male badminton players. A parallel, two-group, stratified randomized controlled design was employed. The intervention lasted 12 weeks, with two sessions per week. All dependent variables were assessed at baseline and after 12 weeks. Following the training period, participants completed sport-specific performance tests, including the 20 m linear sprint, countermovement jump, agility *t*-test, hexagon test, and Y-balance test. On all testing days, participants were instructed to refrain from consuming alcohol or caffeine. Measurements were taken at the same time of day to ensure consistency. Additionally, participants were advised to maintain stable hydration, sleep, and nutritional levels throughout the study.

### 2.2. Participants

Twenty-four well-trained male badminton players with a mean age of 13.5 ± 1.15 years participated in this study (body mass 48.93 ± 9.99 kg, body height 160.07 ± 11.26 cm). Participants were separated by maturity offset with 12 pre-PHV and 12 post-PHV athletes. Participants had a mean training background of 4.40 ± 1.40 years and participated, on average, in 8–10 h of badminton training per week. Each participant provided informed consent prior to the start of the study. Those who had lower extremity, lower-back, or upper extremity muscle-related injury in the 6 months prior to the start of the study were excluded. In addition, all participants regularly in badminton training sessions. All experiments were conducted in accordance with the Declaration of Helsinki and were approved by the Institutional Review Board of Jen-Ai Hospital (approval number: IRB-108-06).

### 2.3. Criterion Measures

Before the first testing day, all participants attended an introductory session, during which they were fully familiarized with the experimental and testing procedures.

### 2.4. Anthropometrics

To calculate biological age, all participants were measured for chronological age, standing height, sitting height, leg length, body mass, and maturity offset (MO) during baseline testing. These variables were used to calculate the maturity offset following the formula proposed by Mirwald et al. [18]. Negative maturity offset values are defined as pre-PHV, while positive values are defined as post-PHV [19].

### 2.5. 20 m Linear Sprint Test

A 20 m linear sprint test was conducted using a Smartspeed Pro timing gate system (Fusion Sport, Boulder, CO, USA). In the standing position, the participants sprinted 20 m when they heard the audio cue. Three trials were performed with 2 min of rest between each. The best maximal running time was measured over 0–20 m. Acceleration was measured over 0–10 m. This test has an ICC greater than 0.9, suggesting high test–retest reliability [20].

### 2.6. Countermovement Jump

The participants stood on a jump mat (*Smart Jump*, *Fusion Sport*, Brisbane, Australia) with both hands placed on hips, and were instructed to perform a countermovement to a depth that would elicit the greatest jump height and maintain fully extended lower limbs throughout the flight period. The flight time was used to estimate the jump height by the formula: gt^2^/8, where *h* is jump height (m), *t* is flight time (s), and *g* is the gravity acceleration (9.81 m·s^−2^) [21]. All participants performed 3 jumps, each separated by 1 min, with the height of each being recorded (centimeters). This test has an ICC greater than 0.9 [22].

### 2.7. Agility t-Test

The agility *t*-test was conducted using a Smartspeed Pro timing gate system (Fusion Sport, Boulder, CO, USA). It was used to determine changes in directions such as right and left sides, forward sprinting, and back-pedaling. The agility *t*-test details and procedures are detailed in Pauole et al. [23]. The test–retest reliability of this test was 0.98 [24]. Three trials were performed with a 1 min rest interval between each trial. The fastest time was recorded for data analysis.

### 2.8. Hexagon Test

The test has been used as a measure of agility and foot quickness. The test began with the participants standing on the tape strip placed in the middle of the hexagon, facing forward in the middle of a hexagon measuring 60 cm per side and with each angle being 120 degrees. With feet together and hips facing forward throughout the test sequence, participants double-leg hopped forward and backward in a clockwise manner over each of the 6 sides of the hexagon, completing 3 sequences. This test has an ICC of 0.93 [25]. Three trials were performed with a 1 min rest interval between each trial. The fastest time was recorded for data analysis.

### 2.9. Y-Balance Test (YBT)

The test involved unilateral lower limb reaches in three directions: anterior (A), postero-medial (PM), and postero-lateral (PL). Participants completed three official trials in each direction. During each trial, participants kept their hands on their hips while reaching with the non-stance leg barefoot, ensuring that the stance heel remained in contact with the ground. The maximum reach distance from the three trials in each direction was recorded for analysis, and all reach distances were normalized to the participant’s leg length. A composite score (%) was calculated using the following formula: [(maximum anterior reach distance + maximum posteromedial reach distance + maximum posterolateral reach distance)/(leg length × 3)] × 100. The test was conducted according to a published protocol and was made nondirectional to calculate the total YBT reach asymmetry. The test–retest reliability of this test was 0.99 [26,27].

### 2.10. Training Program

Every training session consisted of a 10 min dynamic warm-up. After the dynamic warm-up, participants performed the integrated NMT exercise program focusing on the development of whole-body balance ability, coordination ability, lower extremity strength training, core stability training, plyometric training, acceleration, deceleration, and agility training. The 12-week training program consisted of two 40–60 min sessions per week on nonconsecutive days. The selection of exercises was based on a review of the following articles [9,28,29,30,31,32].

The initial 4 weeks focused primarily on skill acquisition to establish a solid training foundation and refine movement techniques. Participants were advised to maintain strong focus and awareness of their movement quality, with particular attention to core stability and the alignment of the hip and knee relative to the foot, ensuring the knee remained positioned over the toe. After the fourth week, the program will progressively increase the intensity and difficulty of the exercises (Table 1). (1) Balance and coordination training advanced from static to dynamic single-leg activities and incorporated unstable surfaces (e.g., BOSU balls, rocker boards). From week 5 onward, a 1 kg medicine ball was introduced to challenge postural control and enhance perturbation response. Badminton specificity: Single-leg balance with contralateral leg movements mimics the split stance and reactive postures used in badminton footwork recovery and lunging actions. (2) Plyometric training progressed from bilateral to unilateral drills and from low to moderate jump heights. By weeks 9–12, box and depth jumps (20 cm) were introduced. Badminton specificity: Jumping patterns (lateral, forward-backward, single-leg) replicate the explosive take-offs and landings seen during rallies, net play, and smashes. (3) Acceleration, deceleration, and agility training evolved from 4-point to 6-point directional change drills, increasing both complexity and speed. Badminton-specific footwork and ladder drills were incorporated throughout. Badminton specificity: These drills emphasize directional agility and mirror on-court movement patterns such as lunges, recovery steps, and split-step initiations. (4) Strength training began with bodyweight exercises and progressed to include Bulgarian bags loaded with 5–10% of body mass from weeks 5 to 12. Badminton specificity: Unilateral lower-limb strengthening (e.g., split squats, lunges, Romanian deadlifts) supports force production during single-leg take-offs and multidirectional changes. (5) Core stability training increased in complexity by incorporating dynamic limb movements and single-leg variations. Repetitions and movement complexity gradually progressed over 12 weeks. Badminton specificity: Rotational and unilateral core challenges replicate trunk control demands during overhead strokes and rapid lateral movements. Coaches implementing these training programs must hold a valid professional certification in athletic or strength and conditioning coaching.

### 2.11. Statistical Analyses

A priori power analyses (G*Power 3.1) indicated that the minimum sample size was 10 participants, which resulted in statistical power values of 0.80 [33]. Descriptive statistics (mean and SD) were calculated for each of the variables. The homogeneity of regression assumption was tested, and the results of Levene’s test of equality indicated that the assumption of homogeneity of variance was supported.

An independent samples *t*-test was performed to test for between-group differences in anthropometric measurements and baseline values of the dependent variables. Paired samples Student’s *t*-test was used to identify differences between pre- and post-test within both the pre-PHV and post-PHV groups. The effect of the maturity group (pre-PHV and post-PHV) on dependent variables was analyzed through an analysis of covariance (ANCOVA). The pre-test values were used as a covariate, the post-test values as the dependent variable, and the maturity status as the independent variable. Effect sizes (ESs) were calculated to estimate the magnitude of differences between pretest and posttest means, divided by their common standard deviation (SD) for the tested variables and interpreted using the following thresholds: <0.2 (trivial); 0.20 to 0.59 (small), 0.60 to 1.19 (moderate), 1.20 to 1.69 (large), and >1.70 (very large) [34]. The level of significance used was set at *p* < 0.05.

## 3. Results

**Figure 1:**
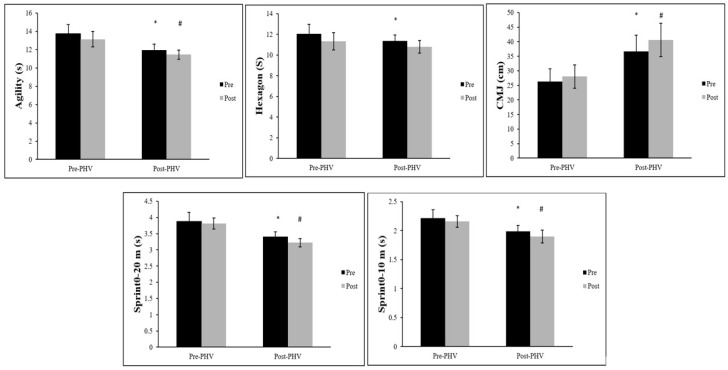
Comparisons of the 10 m sprint, 20 m sprint, CMJ, agility, and hexagon performance tests before and after a 12-week training period for both groups. PHV: Peak height velocity. *: significant between-group difference compared to the pre-PHV group at the pre-test (*p* < 0.05). #: significant between-group difference compared to the pre-PHV group at the post-test (*p* < 0.05).

This study examined the effects of a 12-week integrated NMT program on various sports performance tests, comparing individuals before and after peak height velocity (PHV). Figure 1 presents the mean ± SDs and effect sizes of the 20 m linear sprint, countermovement jump, agility *t*-text, and hexagon test. Following a 12-week training period for both groups, the post-PHV group demonstrated superior performance in sprinting, CMJ, and agility-related tasks compared to the pre-PHV group. Table 2 presents the mean ± SDs anthropometric characteristics for all the participants. Table 3 presents the mean ± SDs and effect sizes of the Y-balance test following the 12-week NMT program for the pre- and post-PHV groups. After completing the training program, most YBT parameters showed significant improvements in both groups. However, the pre-PHV group showed significantly greater improvements in YBT non-directional asymmetry performance than the post-PHV group.

### 3.1. Anthropometric

Table 2 presents the mean ± SDs of the anthropometric characteristics for the pre-PHV and post-PHV groups. The post-PHV group was chronologically older, taller, and heavier than the pre-PHV group (*p* < 0.05).

### 3.2. 20 m Linear Sprint Test

For the 10 m and 20 m sprint times, the pre-PHV group exhibited significantly poorer pre-test values compared to the post-PHV group. After the 12-week training, both the pre-PHV (t = 2.73, effect size = 0.49, *p* < 0.05) and post-PHV (t = 4.26, effect size = 0.86, *p* < 0.05) groups showed significant improvements in 10 m sprint performance. However, after ANCOVA analysis, controlling for pre-test values, the post-PHV group had significantly superior 10 m sprint performance compared to the pre-PHV group (*p* < 0.05).

A similar trend was observed in the 20 m sprint test, where only the post-PHV group (t = 6.25, effect size = 1.35, *p* < 0.05) showed a significant improvement. The pre-PHV group (t = 1.50, effect size = 0.30, *p* > 0.05) did not show a significant improvement. However, after ANCOVA analysis, controlling for pre-test values, the post-PHV group continued to outperform the pre-PHV group (*p* < 0.05).

### 3.3. Countermovement Jump

For the CMJ height, the pre-PHV group exhibited significantly lower pre-test values compared to the post-PHV group. After the 12-week training, both the pre-PHV (t = −3.16, effect size = 0.40, *p* < 0.05) and post-PHV (t = −6.17, effect size = 0.70, *p* < 0.05) groups showed significant improvements in CMJ performance. However, after ANCOVA analysis, controlling for pre-test values, the post-PHV group still demonstrated significantly superior CMJ performance compared to the pre-PHV group (*p* < 0.05) after the 12-week training.

### 3.4. Agility t-Test

For agility performance, the pre-PHV group exhibited significantly poorer pre-test values compared to the post-PHV group. After the 12-week training, both the pre-PHV (t = 4.24, effect size = 0.61, *p* < 0.05) and post-PHV (t = 6.53, effect size = 0.86, *p* < 0.05) groups showed significant improvements in agility performance. However, after ANCOVA analysis, controlling for pre-test values, the post-PHV group still demonstrated significantly superior agility performance compared to the pre-PHV group (*p* < 0.05) after the 12-week training.

### 3.5. Hexagon Test

Regarding the hexagon test, the pre-PHV group exhibited significantly poorer pre-test values compared to the post-PHV group. After the 12-week training, both the pre-PHV (t = 4.81, effect size = 0.81, *p* < 0.05) and post-PHV (t = 6.61, effect size = 1.03, *p* < 0.05) groups showed significant improvements in hexagon performance. However, after ANCOVA analysis, controlling for pre-test values, the post-PHV group did not demonstrate significantly superior hexagon performance compared to the pre-PHV group (*p* > 0.05) after the 12-week training.

### 3.6. Y-Balance Test

For YBT–ANT of the right leg, the pre-PHV group exhibited significantly poorer pre-test values compared to the post-PHV group (*p* < 0.05). There were no significant differences in the YBT-PL and PM of the right leg between the pre-PHV group and the post-PHV group at the pre-test (*p* > 0.05). After the 12-week training, both the pre-PHV (t = −5.85, effect size = 1.23, *p* < 0.05) and post-PHV (t = −2.77, effect size = 1.02, *p* < 0.05) groups showed significant improvement. However, after ANCOVA analysis, controlling for pre-test values, the post-PHV group did not demonstrate significantly superior YBT–ANT performance compared to the pre-PHV group (*p* > 0.05) after the 12-week training.

For YBT–PL and PM of the right leg, after the 12-week training, both the pre-PHV (t = −2.50, effect size = 0.57; t = −3.42, effect size = 0.60, *p* < 0.05) and post-PHV (t = −4.20, effect size = 0.79; t = −4.16, effect size = 1.08, *p* < 0.05) groups showed significant improvement, respectively.

Additionally, for the YBT–composite score of the right leg, the pre-PHV group demonstrated significantly higher pre-test values compared to the post-PHV group (*p* < 0.05). After the 12-week training, both the pre-PHV (t = −7.60, effect size = 0.60, *p* < 0.05) and post-PHV (t = −4.67, effect size = 0.69, *p* < 0.05) groups showed significant improvement. However, after ANCOVA analysis, controlling for pre-test values, the post-PHV group did not demonstrate significantly superior YBT–composite score performance compared to the pre-PHV group (*p* > 0.05) after the 12-week training.

For YBT–ANT of the left leg, the pre-PHV group exhibited significantly poorer pre-test values compared to the post-PHV group (*p* < 0.05). There were no significant differences in the YBT-PL and PM of the left leg between the pre-PHV group and the post-PHV group at the pre-test (*p* > 0.05). After the 12-week training, both the pre-PHV (t = −3.77, effect size = 1.02, *p* < 0.05) and post-PHV (t = −2.95, effect size = 0.87, *p* < 0.05) groups showed significant improvement. However, after ANCOVA analysis, controlling for pre-test values, the post-PHV group did not demonstrate significantly superior YBT–ANT performance compared to the pre-PHV group (*p* > 0.05) after the 12-week training.

For YBT–PL of the left leg, after the 12-week training, both the pre-PHV (t = −2.27, effect size = 0.45, *p* < 0.05) and post-PHV (t = −4.34, effect size = 1.03, *p* < 0.05) groups showed significant improvement. YBT –PM of the left leg, after the 12-week training, the pre-PHV (t = −0.51, effect size = 0.13, *p* > 0.05) did not show a significant improvement, but the post-PHV (t = −2.71, effect size = 0.77, *p* < 0.05) group showed significant improvement. There were no significant differences in the YBT-PL and PM between the pre-PHV group and the post-PHV group at the post-test (*p* > 0.05).

Additionally, for the YBT–composite score of the left leg, the pre-PHV group demonstrated significantly higher pre-test values compared to the post-PHV group (*p* < 0.05). After the 12-week training, both the pre-PHV (t = −3.48, effect size = 0.39, *p* < 0.05) and post-PHV (t = −4.07, effect size = 0.56, *p* < 0.05) groups showed significant improvement. However, after ANCOVA analysis, controlling for pre-test values, the post-PHV group did not demonstrate significantly superior YBT–composite score performance compared to the pre-PHV group (*p* > 0.05) after the 12-week training.

Additionally, only the pre-PHV group showed significant improvements in nondirectional asymmetry magnitude (t = 2.35, effect size = 0.91, *p* < 0.05), while the post-PHV group (t = −0.03, effect size = 0.02, *p* > 0.05) did not. However, after ANCOVA analysis, the pre-PHV group significantly superior YBT–nondirectional asymmetry magnitude performance compared to the post-PHV group (*p* < 0.05) after the 12-week training.

## 4. Discussion

To our knowledge, this is the first study analyzing the impact of 12 weeks (two sessions per week) of integrated NMT, which included strength, core stability, balance, coordination, plyometric training, and agility training, on the athletic performance of pre-PHV and post-PHV badminton players. The results of this study underscore the importance of maturation status in determining how youth athletes respond to integrated NMT interventions. The most important finding was that both pre-PHV and post-PHV groups showed significant improvements across various physical performance tests, although the magnitude and nature of the improvements differed. However, the post-PHV group outperformed the pre-PHV group in sprinting, CMJ, and agility sports performance. It is worth noting that the pre-PHV group exhibited significantly improved YBT-nondirectional asymmetry magnitude performance compared to the post-PHV group.

However, the results of this study align with previous research on integrated NMT programs, although most of these studies were conducted on different groups of youth athletes (e.g., soccer, basketball, tennis) [18,28,30,31,35]. Integrated NMT combines the benefits of balance training, speed, and agility training, plyometric exercises, and coordination drills, all of which help reduce proprioception and central nervous system reaction times, ultimately improving motor performance in athletes [17]. Although numerous studies have investigated the effect of other forms of NMT, the use of single exercise components or combinations of two or more exercise components has also been shown to enhance athletic performance or improve postural control [30,31,35].

While many studies have focused on athletes from other sports like tennis, one study revealed that a 5-week (three sessions per week) NMT program incorporating dynamic warm-up, plyometric training, acceleration, deceleration, and agility drills improved 5, 10, and 20 m, 5-0-5 agility, CMJ, overhead medicine ball throw, and serve velocity in pre-PHV tennis players [31]. Additionally, a 6-week (three sessions per week) integrated NMT program, including dynamic warm-up, jump training, strength training, speed, agility drills, and flexibility, improved speed, agility, abdominal endurance, and single-leg function and balance in 11–16-year-old junior tennis players [28]. In a 10-week (two sessions per week) NMT program focused on strength and agility development, significant improvements were seen in bilateral vertical jump and horizontal jump, as well as explosive strength and 20 m speed. However, no gains were seen in unilateral jumps, COD, or asymmetry in circa-PHV junior tennis players [18].

Female netball athletes aged 11–13 years completed a 6-week (three sessions per week) NMT program that integrated plyometrics and resistance training. As a result, they showed significant improvements in 20 m sprint time, 505 agility time, countermovement jump height, and peak power. Additionally, they demonstrated enhanced movement competency, with increased reach in the anterior and posteromedial directions for both legs, as well as in the posterolateral direction for the left leg only, as measured by the Star Excursion Balance Test [36].

In a similar study, an integrated NMT program (6-week, three sessions per week) on female players (aged 15 years) focused on plyometrics, core strengthening and balance, resistance, and speed training. The use of progressive overload in the program was shown to be more effective in improving performance and movement competency by systematically increasing strength levels. The study found that adolescent athletes were able to improve their single-leg hop distance, vertical jump height, and 9.1 m sprint speed, and demonstrated a reduction in knee valgus and varus torques [12].

However, Panagoulis et al. [37] conducted a study using an 8-week (three sessions per week) integrative neuromuscular strength training program, which initially focused on learning proper exercise techniques before progressing to the development of core strength, hamstring eccentric strength, hip/knee musculature, and dynamic stability. This training also improved 10 and 20 m sprint speed, jumping ability, change of direction, and shooting speed performance in early-adolescent soccer athletes [35]. Similarly, another study focused on young elite soccer players (aged 12–13 years) who trained for 8 weeks (two sessions per week) and compared two training sequences: 4 weeks of balance training followed by 4 weeks of plyometric training (BPT), or the reverse (PBT). Both improved jumping, hopping, sprint acceleration, and balance tests, but BPT resulted in greater gains in the reactive strength index, leg stiffness, the triple hop test, and a trend toward improved Y-Balance test performance compared to PBT [38]. Notably, one study focused on female badminton players (aged 16–19 years) and demonstrated that 8 weeks (four sessions per week) of integrated NMT, which included balance ability, coordination, plyometric training, speed and agility, and core stability training, significantly improved functional movement screening scores, vertical jump, balance ability, strength, and specific athletic performance [17]. Based on the aforementioned studies, it can be concluded that neuromuscular training for post-PHV individuals may require a longer training period.

Furthermore, the results of this study are consistent with those of several other studies, showing that post-PHV individuals exhibit greater improvements in sports performances following integrated NMT compared to pre-PHV individuals. For example, a 4-week (two sessions per week) NMT focusing on whole-body resistance training, core muscle exercise, and plyometrics has been shown to improve movement competency in the back-squat assessment for both pre-PHV and post-PHV male athletes [13]. However, another study suggested that a 6-week (two sessions per week) NMT focused on changes of direction training improved Y-balance, horizontal jump, and changes of direction without the ball in male pre-PHV and post-PHV soccer players, with greater improvements observed in the post-PHV group [14]. In a longer 12-week (two sessions per week) NMT, the results revealed that only the post-PHV training group significantly increased absolute isometric peak force and peak rate of force development within the isometric midthigh pull after training. The pre-PHV training group showed a small increase in the reactive strength index modified, while the post-PHV training group showed a moderate increase, suggesting that post-PHV athletes underwent more significant force-related adaptations. This group also exhibited a greater increase in CMJ height, likely attributed to these more pronounced force-related adaptations [39].

This notion is also supported by Radnor et al. [40], who suggested that adolescents tend to have a greater rate of force development compared to preadolescents due to less agonist-antagonist co-contraction. Furthermore, more mature individuals demonstrate greater efficiency in recruiting high-threshold type II motor units, which contributes to heightened explosive force production during stretch-shortening cycle activities [41]. This underscores the importance of maturation in influencing the effectiveness of strength and power training programs, particularly in terms of force production and explosive movements.

Relevant literature has shown that an NMT program significantly increases back squat strength, which is crucial for maintaining stable knee joint extension and flexion during high-speed exercises [35]. This improvement in strength helps reduce the risk of knee valgus in lower limb biomechanics, further supporting the benefits of NMT for enhancing athletic performance [12]. In addition to improving muscle strength and agility, NMT also contributes to maintaining proper alignment of the trunk, hip, knee, and ankle joints. Correct joint alignment is particularly important because improper knee angles during movement can elevate the risk of anterior cruciate ligament (ACL) injuries. Building on this, plyometric training, alongside balance and coordination training, can significantly enhance neuromuscular function in adolescents. During this developmental stage, improvements in athletic performance are primarily driven by neuromuscular adaptations rather than muscle hypertrophy, particularly through the recruitment of additional motor units [37,42]. Research indicates that just four weeks of plyometric training can significantly improve sports performance in youth athletes (aged 8–12 years), with measurable gains in the reactive strength index and leg stiffness, both of which are linked to these performance improvements [23,40]. Moreover, strength and core stability training play a vital role in enhancing performance in agility and jumping. Core muscle control is essential for maintaining stability during dynamic lower-body movements in sports. Without sufficient core strength, the stability of the lower limbs is compromised, which in turn hinders efficient force transmission and connection through the distal limbs [43]. This lack of stability can lead to issues extending from the lower back down to the ankle [44]. Therefore, NMT not only enhances athletic performance but also plays a crucial role in injury prevention, especially ACL tears, by promoting safer, more controlled movements during high-intensity activities such as those performed on the badminton court.

Notably, badminton is a sport in which limb asymmetries often arise due to the repetitive use of the dominant hand and leg [45]. Additionally, during step-forward lunges and jump lunges, the dominant leg has been found to generate greater force [46]. As a result, structural asymmetries between the lower limbs are commonly reported in badminton players [47]. The findings of this study indicate that both the pre-PHV and post-PHV groups exhibited bilateral asymmetry in the YBT test. However, after NMT training, both groups showed improvements in the YBT–composite score, with only the pre-PHV group demonstrating greater improvements in nondirectional asymmetry magnitude and showing significantly better improvement than the post-PHV group.

Based on our results, maturity seems to have a considerable influence on postural control. It might be that deficits in balance performance contribute to the phenomenon of adolescent awkwardness [48]. Although this study did not assess lower limb strength, previous research by Lee et al. (2014) [30] observed a positive correlation between lower limb strength and reach distance in all three directions of the YBT in women. Additionally, a strong relationship was found between knee-flexor strength and performance across all three directions, which was attributed to the dynamic postural control required during the YBT. The study also highlighted that greater hip flexion is necessary to achieve longer reach distances, thus increasing the demand for hip-extensor strength to maintain postural control. These findings suggest that strength, particularly in the lower limbs, may play a key role in improving YBT performance and asymmetry [49].

In line with this, the present study also investigated the effects of an 8-week body-weight neuromuscular training (NMT) program (two sessions per week), which focused on strength and agility development, in adult female basketball players. The results showed significant improvements in both the lower limbs’ posteromedial and posterolateral reach, as well as composite YBT scores. However, no significant changes were observed in the anterior reach direction [30]. Similarly, an 8-week prevention program that included lower limb strength and plyometric training, along with joint and core stability exercises, was shown to improve lower limb Y-balance stability in young volleyball players [50].

Therefore, it is speculated that for post-PHV badminton players, relying solely on NMT may have limited effects on improving the Y-balance nondirectional asymmetry magnitude. It is likely that additional unilateral strength training, with appropriate intensity and volume, may be necessary to better address the asymmetry issues in the Y-balance test for both legs. In addition, training should incorporate progressive resistance training, including free weights and eccentric loading, to promote strength, power, and injury resilience. High-intensity plyometrics, acceleration/deceleration drills, and sport-specific agility training should be prioritized to enhance performance outcomes. However, continued monitoring of asymmetry and mobility restrictions is essential, particularly in high-risk areas such as the hip, knee, and ankle joints, to inform corrective interventions.

For pre-PHV badminton players, training should prioritize the development of fundamental movement skills, motor control, and neuromuscular coordination, as this stage corresponds to rapid neurological maturation. Emphasis should be placed on bodyweight and low-load resistance exercises that target balance, postural alignment, and movement technique, aiming to enhance movement efficiency while minimizing the risk of asymmetries or compensatory patterns.

To further improve postural control and joint stability, training should incorporate proprioceptive exercises and dynamic balance activities. In addition, a moderate volume of plyometric and multidirectional agility drills can be introduced to stimulate reactive strength and movement adaptability.

## 5. Limitations and Future Research

The study focused only on badminton players, and the results may not be directly applicable to athletes in other sports. It would be beneficial to replicate this study in different sports to examine whether similar trends occur across various athletic populations. Furthermore, the absence of a non-intervention control group and the lack of control for individual variations in hormonal maturation represent methodological limitations that could influence the interpretation of training effects.

In conclusion, this study highlights the critical role of maturation status in determining the response to training interventions in youth athletes. Tailoring training programs to an athlete’s developmental stage can optimize performance gains and ensure more effective and sustainable outcomes. Further research is needed to explore the long-term effects and broader applicability of these findings across different sports and populations.

## Figures and Tables

**Table 1:** Description of the multicomponent neuromuscular training (NMT) program.

Component/wk	Balance, Coordination	Plyometrics Training	Acceleration, Deceleration, Agility	Strength Training	Core Stability Training
1–4 (wk)	Double- and single-leg balance on unstable surfaces hold Single-leg stand on unstable surfaces with the contralateral leg balancing from 45° flexion to 45° extension Double- and single-leg heel–toe raises on unstable surfaces Double- and single-leg balance on unstable surfaces to catch the tennis ball # Each exercise was held in position for 30 s per leg. Perform all exercises as one set. Complete a total of 3 sets, resting for 1 min between each.	Step hold Wall jump Tuck jump Squat jump Lateral jump and hold Bounding drills (single-leg and double-leg) # Each exercise was performed for 10 reps. Perform all exercises as one set. Complete a total of 3 sets, resting for 1 min between each.	Ladder: various patterns (e.g., forward, lateral, side to side, crossover quick steps) Badminton 4-point change of direction # Each exercise was performed for 10 s. Perform all exercises as one set. Complete a total of 3 sets, resting for 1 min between each.	Split squat Front lunges Backward lunge Side lunge Double-legged calf raises on step Single-legged Romanian deadlift # Each exercise was performed for 20 reps (10 each leg or side). Perform all exercises as one set. Complete a total of 3 sets, resting for 1 min between each.	Plank on elbows Side bridge Double leg bridge Abdominal crunches Back hyperextension on the ground # Each exercise was held in position for 30 s. Perform all exercises as one set. Complete a total of 3 sets, resting for 1 min between each.
5–8 (wk)	Double- and single-leg balance on unstable surfaces Double- and single-leg heel–toe raises on unstable surfaces Single-leg stand on unstable surfaces with the contralateral leg balancing from 45° flexion to 45° extension # Each exercise was held in position for 30 s per leg. Perform all exercises as one set. Complete a total of 3 sets, resting for 1 min between each, and was performed with a 1 kg medicine ball.	Barrier jump side to side Barrier jump forward–backward Broad jump Single tuck jump with a soft landing Scissor Jumps # Each exercise was performed for 10 reps. Perform all exercises as one set. Complete a total of 3 sets, resting for 1 min between each.	Ladder: various patterns (e.g., forward, lateral, side to side, crossover quick steps) Badminton 4-point change of direction # Each exercise was performed for 10 s. Perform all exercises as one set. Complete a total of 3 sets, resting for 1 min between each.	Split squat Front lunge Backward lunge Side lunge Double-legged calf raises on step Single-legged Romanian deadlift # Each exercise with an additional Bulgarian bag (loaded with 5% of body mass) # Each exercise was performed for 20 reps (10 each leg or side). Perform all exercises as one set. Complete a total of 3 sets, resting for 1 min between each.	Plank on elbows, alternate 1 leg lift Plank on elbows while raising the arm and the opposite leg Side bridge while raising the arm and leg Single leg bridge # Each exercise was performed for 30 reps per leg or side. Perform all exercises as one set. Complete a total of 3 sets, resting for 1 min between each.
9–12 (wk)	Double- and single-leg front jumps on unstable surfaces Double- and single-leg lateral jumps on unstable surfaces # Each exercise was performed for 20 reps or 10 reps per leg. Perform all exercises as one set. Complete a total of 3 sets, resting for 1 min between each, and was performed with a 1 kg medicine ball # Balance exercises using stability ball, rocker boards, bosu, and medicine ball instrumentation.	Triple broad jump Single-leg triple hop Box jumps and depth jumps height of 20 cm # Each exercise was performed for 10 reps. Perform all exercises as one set. Complete a total of 3 sets, resting for 1 min between each.	Ladder: various patterns (e.g., high knees, lateral quick steps) Badminton 6-point change of direction # Each exercise was performed for 10 s. Perform all exercises as one set. Complete a total of 3 sets, resting for 1 min between each.	Split squat Front lunge Backward lunge Side lunge Double-legged calf raises on step Single-legged Romanian deadlift # Each exercise with an additional Bulgarian bag (loaded with 10% of body mass) # Each exercise was performed for 20 reps (10 each leg or side). Perform all exercises as one set. Complete a total of 3 sets, resting for 1 min between each.	Plank on elbows while raising the arm and the opposite leg Side bridge while raising the arm and leg Plank with Knee to opposite elbow and ups-down side Single leg hip lift # Each exercise was performed for 30 reps per leg or side. Perform all exercises as one set. Complete a total of 3 sets, resting for 1 min between each.

Reps = repetitions; s = seconds.

**Table 2:** Anthropometric characteristics of the pre-PHV and post-PHV groups.

	Pre-PHV	Post-PHV
Chronological age (years)	12.67 ± 0.49	14.67 ± 0.49 *
Standing height (cm)	154.33 ± 8.11	170.75 ± 6.24 *
Sitting height (cm)	80.17 ± 3.18	87.67 ± 3.37 *
Leg length (cm)	73.58 ± 4.98	82.17 ± 4.42 *
Body mass (kg)	44.50 ± 7.59	56.76 ± 6.54 *
Maturity offset	−1.21 ± 0.45	0.76 ± 0.58 *

PHV: peak height velocity. *: significant difference with the pre-PHV group (*p* < 0.05).

**Table 3:** Comparisons of the YBT performance tests before and after a 12-week training period for both groups.

	Pre-PHV			Post-PHV		
	Pre	Post	Effect Size	Pre	Post	Effect Size
Right leg						
YBT-ANT (cm)	58.67 ± 5.72	65.17 ± 4.80 ^+^	1.23	63.92 ± 3.73 *	67.67 ± 3.63 ^+^	1.02
YBT-PL (cm)	101.33 ± 4.92	104.17 ± 4.99 ^+^	0.57	103.17 ± 3.66	106.83 ± 5.46 ^+^	0.79
YBT-PM (cm)	97.92 ± 8.25	102.42 ± 6.7 ^+^	0.60	97.75 ± 5.26	104.5 ± 7.08 ^+^	1.08
Composite score (%)	116.38 ± 10.42	122.43 ± 9.78 ^+^	0.60	107.21 ± 7.39 *	112.47 ± 7.84 ^+^	0.69
Left leg						
YBT-ANT (cm)	60.08 ± 5.28	66.08 ± 6.46 ^+^	1.02	65.33 ± 4.64 *	69.33 ± 4.56 ^+^	0.87
YBT-PL (cm)	102.83 ± 7.04	105.75 ± 5.91 ^+^	0.45	101.92 ± 4.64	107.5 ± 6.14 ^+^	1.03
YBT-PM (cm)	101.42 ± 6.13	102.08 ± 4.23	0.13	100.33 ± 4.56	103.83 ± 4.57 ^+^	0.77
Composite score (%)	119.38 ± 11.94	123.50 ± 9.13 ^+^	0.39	109.63 ± 7.96 *	114.37 ± 8.88 ^+^	0.56
Nondirectional asymmetry magnitude (cm)	7.75 ± 7.25	2.67 ± 3.02 ^+^	0.91	6.58 ± 5.90	6.67 ± 5.45 ^#^	0.02

PHV: peak height velocity. YBT–ANT, PL, PM: anterior; posterolateral; posteromedial. *: significant between-group difference compared to the pre-PHV group at the pre-test (*p* < 0.05). #: significant between-group difference compared to the pre-PHV group at the post-test (*p* < 0.05). +: statistically significant difference between pre-test (*p* < 0.05).

## Data Availability

Data are contained within the article.
